# A reference quality, fully annotated diploid genome from a Saudi individual

**DOI:** 10.1038/s41597-024-04121-2

**Published:** 2024-11-23

**Authors:** Maxat Kulmanov, Rund Tawfiq, Yang Liu, Hatoon Al Ali, Marwa Abdelhakim, Mohammed Alarawi, Hind Aldakhil, Dana Alhattab, Ebtehal A. Alsolme, Azza Althagafi, Angel Angelov, Salim Bougouffa, Patrick Driguez, Changsook Park, Alexander Putra, Ana M. Reyes-Ramos, Charlotte A. E. Hauser, Ming Sin Cheung, Malak S. Abedalthagafi, Robert Hoehndorf

**Affiliations:** 1 Computational Bioscience Research Center (CBRC), King Abdullah University of Science and Technology, Thuwal, Saudi Arabia; 2 KAUST Center of Excellence for Smart Health (KCSH), King Abdullah University of Science and Technology, 4700 KAUST, 23955 Thuwal, Saudi Arabia; 3 KAUST Center of Excellence for Generative AI, King Abdullah University of Sciene and Technology, 4700 KAUST, 23955 Thuwal, Saudi Arabia; 4 Biological and Environmental Sciences & Engineering (BESE) Division, King Abdullah University of Science and Technology, Thuwal, Saudi Arabia; 5 Computer, Electrical and Mathematical Sciences & Engineering (CEMSE) Division, King Abdullah University of Science and Technology, Thuwal, Saudi Arabia; 6 Laboratory for Nanomedicine, Biological and Environmental Science & Engineering (BESE) Division, King Abdullah University of Science and Technology (KAUST), Thuwal, Saudi Arabia; 7 Genomic and Precision Medicine Department, King Fahad Medical City, Riyadh, Saudi Arabia; 8 Computer Science Department, College of Computers and Information Technology, Taif University, Taif, Saudi Arabia; 9 Core Labs, King Abdullah University of Science and Technology (KAUST), 4700 KAUST, 23955 Thuwal, Makkah Saudi Arabia; 10 Max Planck Institute for Biology of Ageing, Cologne, Germany; 11 Institute of Health Care Engineering with European Testing Center of Medical Devices, Graz University of Technology, Stremayrgasse 16/II, 8010 Graz, Austria; 12 Department of Pathology and Laboratory Medicine, Emory School of Medicine, Atlanta, GA USA; 13 King Salman Center for Disability Research, Riyadh, Saudi Arabia; 14 Present Address: Computer, Electrical and Mathematical Sciences & Engineering (CEMSE) Division, King Abdullah University of Science and Technology, Thuwal, Saudi Arabia

**Keywords:** Genome informatics, Genomics

## Abstract

We have used multiple sequencing approaches to sequence the genome of a volunteer from Saudi Arabia. We use the resulting data to generate a *de novo* assembly of the genome, and use different computational approaches to refine the assembly. As a consequence, we provide a contiguous assembly of the complete genome of an individual from Saudi Arabia for all chromosomes except chromosome Y, and label this assembly KSA001. We transferred genome annotations from reference genomes to fully annotate KSA001, and we make all primary sequencing data, the assembly, and the genome annotations freely available in public databases using the FAIR data principles. KSA001 is the first telomere-to-telomere-assembled genome from a Saudi individual that is freely available for any purpose.

## Background & Summary

The first complete, or almost complete, sequence of a human genome was made available in 2020 and published in 2022^1^, based on the functionally haploid cell line CHM13. Since then, several human genome assemblies were published^2,3^, including the diploid genome sequences of 47 individuals^4^. The availability of these genomes is driven both by advances in sequencing technology which make it possible to sequence more accurate and longer reads, and advances in assembly and read mapping algorithms^5–8^ which can efficiently assemble and map sequence reads obtained from different sequencing technologies and of different quality, as well as assemble complex regions of genomes.

The availability of cheap, fast, and accurate sequencing technologies now enables sequencing of multiple genomes from diverse populations in order to understand their genetic variability. For each population, it also becomes possible to develop computational resources and databases that capture their diversity. Creating population-specific computational resources can serve as the foundation for bioinformatics workflows and thereby improve the accuracy of genomic analyses within a population as well as when comparing results between multiple different populations^9^.

Reference genomes in particular are a foundation of workflows that analyze genomic data, and it is well-known that current reference genomes exhibit population bias^10^ that may affect analyses built on them^9^. Specifically, population-specific structural variants will likely not be included in reference genomes that are not derived from the same population, and, consequently, sequencing reads may not be aligned accurately when the corresponding genomic region is missing from the reference genome; this can affect the success of identifying disease-causing variants or pre-disposing variants for common diseases within the population.

In the Middle East, the Qatari genome project^11^ and the United Arab Emirates population genome project^12^ aimed to address this challenge by producing a population-specific reference genome that includes as major alleles the most frequent ones identified within their respective populations. However, many populations in the Middle East have historically been organized in tribal structures where marriages occur predominantly within a tribe^13^ leading to several populations that were isolated for a period of time and therefore exhibit different genetic structure^14^. Additionally, the tribal structure also resulted in a relatively high prevalence of consanguineous marriages and consequently homozygosity and Mendelian diseases^15^. No single reference genome can fully capture the genetic diversity found in different populations within the Middle East.

Although there are many genetic studies of ancient and current populations of the Middle East^16–19^, only little genomic data from the Middle East is publicly available, or data that is available can only be used under prohibitive licenses, not be shared publicly, or not use commercially. However, public availability and permissive licences are crucial for resources that need to be shared and utilized broadly, in particular for reference genomes. To further ensure broad usability, resources that underlie common analyses and workflows should be Findable, Accessible, Interoperable, and Reusable (FAIR)^20^.

We sequenced the genome of a female volunteer from Saudi Arabia who consented to make her genomic data public and freely available. We used three different sequencing strategies based on long and short reads. The first draft genome was created based on a *de novo* genome assembly using these reads. The assembly was further refined using the CHM13 genome^1^ resulting in the most complete publicly available genome sequence of a Saudi individual so far. We use a variety of tools to fully annotate this genome, either transferring information from public databases or using methods from bioinformatics to identify functional elements (genes, regulatory regions) using the genome sequence directly. Our sequencing, assembly, and annotation effort resulted in a reference-quality personal genome from a Saudi individual which we label KSA001.

The assembled genome sequence of KSA001, the primary sequencing data, and the workflows used to construct the genome sequence are freely available on https://github.com/bio-ontology-research-group/KSA001. The sequence reads and assembly are further available in public sequence databases, and the genome is available in standard formats, making this the first telomere-to-telomere-assembled genome from the Arabian peninsula. We provide genome annotations for KSA001 and make a variant calling workflow using KSA001 freely available as well to enable its immediate use in molecular genetics studies.

We make this genome publicly available following the FAIR principles^20^. The genome is findable as it is deposited in repositories containing genome assemblies, and accessible through common protocols and data download utilities. It is also interoperable as we use standard formats used across bioinformatics, and it is reusable as we make the primary data available as well as a description of the workflow that led to the assembled genome. The quality of the assembly and the set of genome annotations we make available, but in particular its free availability, allows KSA001 to be shared and used as a reference genome for genomic studies in Saudi Arabia and the Middle East. We demonstrated that KSA001 can be used for variant calling and therefore can contribute to the success of diagnostic or prognostic genomic methods.

## Methods

### Recruitment

One volunteer provided the sample for KSA001, and the volunteer’s parents also donated samples. The donors provided informed consent for the collection of blood, DNA sequencing, and for making all data public. The criteria for selecting the donor were Saudi nationality with tribal origin, over 18 years old, and the ability to provide informed consent.

The study and recruitment process were advertised in the research center at King Fahad Medical City between 2022 and 2023 and were overseen by Dr. Malak Abedalthagafi. This process included detailed bilingual consent (Arabic and English) and extensive counseling. The donors voluntarily agreed to donate their anonymous samples, with written informed consent obtained from all participants, allowing the data and analyses from the genome sequencing to be made public. The parents of the individual designated as KSA001 agreed to be part of the study and were provided with detailed consent forms and counseling.

### DNA extraction

DNA was extracted using two methods. Ultra high molecular weight DNA (uHMW) was isolated from fresh blood samples using New England Biolabs (NEB) Monarch High Molecular Weight (HMW) DNA isolation kit following manufacturer’s protocol (New England Biolabs, UK) with modification, agitation was set at 700 rpm during the lysis step. DNA was kept at 4 °C until library preparation for long-read sequencing using the PacBio and Oxford nanopore sequencing platforms.

For short read sequencing of KSA001, we isolated donor DNA using the DNeasy Blood & Tissue kit (Qiagen) following the manufacturer’s instructions. DNA was kept at −20 °C until library preparation.

For the parents of KSA001, we extracted genomic DNA from blood samples of the parents of KSA001 using the DNeasy Blood & Tissue Kit (Qiagen, Hilden, Germany). Per the manufacturer’s instructions, we placed 200 *μ*L of blood in a 1.5 mL microcentrifuge tube and mixed it with 20 *μ*L of proteinase K and 200 *μ*L of Buffer AL. We vortexed the sample for 15 seconds and incubated it at 56 °C for 10 minutes to allow complete lysis. We followed the consequent steps in the protocol, which consisted of DNA binding to the silica membrane and washing to remove residual contaminants and proteins. Finally, we eluted the DNA, where we placed the DNeasy Mini spin column in a clean 1.5 mL microcentrifuge tube and pipetted 200 *μ*L of Buffer AE directly onto the membrane. After incubating at room temperature for 1 minute, we centrifuged the column at 6,000 x g for 1 minute.

### Sequencing library preparation and sequencing

We used three platforms for sequencing the extracted DNA: Illumina NovaSeq 6000 (Illumina, San Diego, USA), PacBio Sequel II (Pacific Biosciences, Menlo Park, USA), and Oxford Nanopore PromethION (Oxford Nanopore Technologies, Oxford, UK).

We used 100 ng of genomic DNA (gDNA) as an input to construct a whole genome library to be sequenced using the NovaSeq 6000 platform. The DNA was mechanically sheared with Covaris (Covaris, Woburn, USA) and converted to a sequence-ready library using the TruSeq DNA Nano Library Kit (Illumina, San Diego, USA). Subsequently, the library was quantified using Qubit high-sensitivity dsDNA Assays (model Q33230, ThermoFisher Scientific, Waltham, USA), and the quality control was performed using an Agilent Bioanalyzer 2100 (Agilent Technologies, Santa Clara, USA).

The KSA001 sample was sequenced on two lanes of an SP flowcell with a read length of 2 × 150 bp in paired-end format, which generated 311 GB of data, resulting in an estimated 100x coverage. For the parents of KSA001, we sequenced both libraries on a NovaSeq 6000 system (Illumina, USA) using an SP flowcell (2 × 150 cycles), with each sample sequenced on a single lane. The genome coverage was approximately 23× and 29× for maternal and paternal samples, respectively.

7.5 *μ*g of high molecular weight gDNA was sheared with Megaruptor 3 (Diagenode, Denville, USA) to the size range of 15-20 kb. SMRTbell was prepared with HiFi Express Template prep kit 2.0 (102-088-900), and size-selected with the PippinHT System (Sage Science HTP0001). Finally, SMRTbell QC was assessed with Qubit dsDNA High Sensitivity (model Q33230; ThermoFisher Scientific, Waltham, USA) and FEMTO Pulse (Inc. P-0003-0817; Agilent Technologies, Santa Clara, USA). Sequencing of SMRTbell was set up on PacBio Sequel II system with Sequel II Binding kit 2.2 (101-894-200), Sequel II Sequencing Kit 2.0 (101-820-200), and SMRTcell 8M Tray (101-389-001), according to conditions specified in SMRTlink with 30 hour movie times, 2 hour pre-extension time, and adaptive loading mode.

For Nanopore sequencing, two libraries were prepared using the Ultra-Long Sequencing Kit (SQK-ULK001) and its recommended protocol from Oxford Nanopore Technologies (Oxford Nanopore Technologies, Oxford, UK). 30 *μ*g of uHMW gDNA was used as input for each library. A PromethION flowcell (FLO-PRO002) was primed and prepared according to the same protocol and 75 *μ*l of a sequencing library was loaded. 24 hours after the first sequencing run, a nuclease flush and priming step were performed according to the protocol, and an additional 75 *μ*l of library was loaded before starting another sequencing run. This process was repeated until each library was loaded three times. Basecalling was performed using Guppy v5.1.13 with the super-accurate basecalling model. The entire process is repeated with the second prepared library and another PromethION flowcell.

We employed the Dovetail Omni-C protocol (Dovetail Genomics, USA) to construct Hi-C libraries, a widely adopted method for interrogating chromatin interactions on a genome-wide scale. We initiated the Omni-C Proximity Ligation Assay protocol using 1 ml of fresh whole human blood collected in EDTA-coated tubes as an anticoagulant. The sample was processed to reach a cell density of 1 million cells/mL as an input that then crosslinked using formaldehyde. This process preserves the native chromatin structure, allowing us to work with genomic DNA in its original nuclear context. After cell lysis, we captured chromatin using Chromatin Capture Beads and fragmented it through in situ nuclease digestion. We then performed proximity ligation using an Intra-Aggregate Ligation Enzyme Mix, resulting in chimeric DNA molecules that represent the original three-dimensional chromatin interactions. Following DNA purification and size selection, we quantified the purified DNA using Qubit fluorometry and used 150 ng as input for library preparation. This DNA represents crosslinked, proximity-ligated genomic DNA fragments. The library was generated through end repair, adapter ligation, and index PCR steps. Finally, we subjected the library to a ligation capture step using the Dovetail™ Primer Set for Illumina and amplified it by PCR to generate the final Omni-C library. This approach allowed us to capture and analyze genome-wide chromatin interactions from our blood samples, providing insights into the three-dimensional organization of the genome.

### Assembly

We used PacBio HiFi reads and the ONT ultra-long reads together with Hi-C or reads from the parents to resolve the haplotypes. We applied Hifiasm v0.19.0-r534^21^ and Verkko (commit 508efb)^22^ in Hi-C and Trio modes to generate four haplotype-resolved assemblies. Table 1 shows basic statistics of the sequencing data. We evaluated the quality of the assemblies using Merqury^23^ with *k*-mer size of 21. Table 2 provides statistics on each of our assemblies. We use the Verkko’s Trio assembly as our primary diploid assembly because it resulted in highest sequence length of the shortest contig at 50% (N50) and Quality Value (QV) score. Trio mode assemblies also enable us to determine the maternal and paternal haplotypes.

**Table 1:** Sequencing reads statistics.

Sequencing Technology	Number of Reads	Avg. Read length	GC(%)	Coverage
**Illumina**	2,061,737,744	151	41.3	100x
**PacBio**	8,994,317	17,657	40.6	51x
**Oxford Nanopore**	1,863,431	51,076	40.3	30x
**Hi-C**	729,360,320	151	45.6	35x
**Illumina (Parent 1)**	477,854,908	151	40.1	23x
**Illumina (Parent 2)**	602,505,048	151	40.6	29x

**Table 2:** Assembly statistics and quality.

Tool	Maternal					Paternal				
	Scaffolds	Gaps	N50	Length	QV	Scaffolds	Gaps	N50	Length	QV
**Hifiasm - HiC**	728	0	104.145 MB	3062.204 MB	66.0647	626	0	105.218 MB	3027.886 MB	66.4196
**Hifiasm - Trio**	494	0	106.330 MB	3036.153 MB	65.5969	701	0	99.993 MB	3068.265 MB	66.8000
**Verkko - HiC**	45	11	136.125 MB	3031.626 MB	65.2061	75	11	135.417 MB	3008.205 MB	65.6895
**Verkko - Trio**	92	18	145.092 MB	3026.057 MB	**67.2079**	173	14	136.125 MB	3029.708 MB	63.6325

### Refinement of assembly

After the initial assembly process, we aligned the contigs to CHM13 using minimap2^24^ and manually inspected the gaps that were not linked in previous steps. We found that most of them are in centromeres which contain highly repetitive regions and are generally difficult to align and assemble^25,26^. Using the alignments, we placed the assembly scaffolds into chromosomes. We found that 18 chromosomes completely align to one scaffold in our assembly and chromosomes 13, 15, 19, 21, X map to 2 or 3 scaffolds with gaps around highly repetitive centromeric regions. After this step, maternal and paternal haplotypes had 25 and 23 gaps, respectively.

We performed an additional assembly using the ultra-long ONT reads only with the Flye assembler (2.9.1)^27^ and used MaSuRCA Samba (v4.1.0)^28^ to close the gaps using the ONT based assembly. After this step, the maternal haplotype had 18 gaps and the paternal haplotype had 17 gaps left. We also used the Flye assembly to derive a circular contig with two copies of the mitochondrial genome. Furthermore, we polished the mitochondrial genome from the ONT-based assembly using NextPolish (v1.4.1)^29^ guided by quality-filtered Illumina reads.

We generated a haploid assembly by merging both haplotypes and selecting the best chromosome based on its QV score. We computed QV scores using Merqury^23^ with *k*-mer size of 21 from both Illumina short reads and PacBio HiFi reads. Then, we selected chromosomes with higher QV scores for our haploid KSA001 assembly. We used the BioPython library version 1.81^30^ to generate the haploid assembly using custom code that is available on the project’s code repository. In the haploid KSA001 genome, 15 chromosomes (containing 2,098,976,917 bp) and the mitochondrial genome (16,567 bp) were derived from the maternal haplotype, and 7 chromosomes (containing 959,396,833 bp) were derived from the paternal haplotype. The haploid KSA001 has 12 gaps in total in chromosomes 7 (2 gaps), 9 (1 gap), 13 (2 gaps), 15 (2 gaps), 21 (1 gap), 22 (1 gap), and X (3 gaps). We compared to CHM13 and found that all the gaps are around the centromeric regions with highly repetitive genome sequence.

### Genome annotation

We used the Liftoff tool (v1.6.1)^31^ to transfer gene annotations from CHM13 (v2.0) and GRCh38 (v40). We lifted over UCSC GENCODEv35 CAT/Liftoff v2 gene annotations from CHM13 and UCSC KnownGene, UCSC RefSeq and NCBI RefSeq from GRCh38.

To use KSA001 in downstream applications, we generated chain files between KSA001 (v1.0.0) and both CHM13 (v2.0) and GRCh38 (v40) using minimap2 (v.2.26)^24^ together with ChainTools^32^, rustybam^33^ and paf2chain^34^. Furthermore, we used BCFtools liftover^35^ to transfer the dbSNP (v156) and gnomAD annotation from GRCh38 to KSA001.

### Ethical approval

This work was approved by the Institutional Review Board (IRB) at the Faculty of Medicine, King Fahad Medical City (KFMC) under approval number 22-037, and by the Institutional Bioethics Committee (IBEC) at King Abdullah University of Science and Technology (KAUST) under approval number 22IBEC023. The approval covers the recruitment of one Saudi individual with at least three generations of tribal roots in Saudi Arabia to provide a blood sample. The approved protocol specifies that this sample will be sequenced using multiple sequencing technologies, processed with bioinformatics tools, and published without access restrictions for use in bioinformatics workflows. Additionally, the approval includes the recruitment of 10 more Saudi individuals to provide blood samples for sequencing, with their data also made freely available.

The consent forms were available in both English and Arabic. Participants were recruited by Dr. Malak Abedalthagafi, who is the Principal Investigator (PI) for the approved IRB protocol at King Fahad Medical City. The consent for volunteer KSA001 was co-signed by two witnesses, including the IRB chairperson. Dr. Abedalthagafi personally obtained consent from the volunteers, who participated without any payment or compensation and were recruited from research clinic care. The consent was signed in the presence of two witnesses. There is no known professional or personal relationship between the authors and the participants, except for Dr. Malak Abedalthagafi, who ensured that participants were fully aware of the risks and were competent to understand them. As part of the consent process, participants underwent genetic counseling, as required by the IRB protocol.

None of the other authors were involved in the ethical approval processes at KFMC or KAUST. Robert Hoehndorf was a member of the IBEC at KAUST at the time of approval but did not participate in the review or discussion of the research protocol, and was not present during its discussion.

## Data Records

Data, supporting information, and a description of the assembly workflow are available at https://github.com/bio-ontology-research-group/KSA001. Sequencing reads are available on the Sequence Read Archive under accession numbers SRR21927836^36^, SRR21927835^37^, SRR21927834^38^, SRR21927833^39^, SRR29122519^40^, and SRR29092487^41^, and collected under the project SRP402943 (NCBI BioProject PRJNA891101). Assembled genomes for maternal and paternal haplotypes are available at NCBI Datasets Genomes under accession numbers GCA_037177635.1^42^ and GCA_037177555.1^43^, respectively.

## Technical Validation

### Comparison to CHM13 and GRCh38

We first annotated the KSA001 genome by transferring genome features from CHM13 to KSA001 using the Liftoff (v1.6.1) tool^31^. Liftoff successfully mapped 62,351 genes out of 63,494 genes of all types (98%, Table 3) to KSA001. When we further considered overlapping genome feature annotations, 1,072 additional genes were mapped. By default, Liftoff considers a gene as mapped when it satisfies at least 50% of alignment coverage and sequence identity. Most annotated genes were mapped with sequence identity higher than 99%. Table 4 provides the summary of mapped genes by the sequence identity and Table 5 provides a summary of all mapped genome features. Out of 19,969 protein coding genes, the Proline Rich 32 (PRR32, ENTREZ:100130613) gene is completely missing from KSA001 in comparison to genes in CHM13.

**Table 3:** LiftOff annotation statistics.

	Genes			Transcripts	
	Total	Protein coding	Non-coding	Total number	Protein coding
**CHM13**	63,494	19,969	43,525	234,903	156,412
**KSA001**	63,421	19,968	43,453	233,494	155,918

**Table 4:** LiftOff annotation statistics. Number of mapped genes from CHM13 grouped by sequence identity.

Genes	100%	≥99%	≥95%	≥90%	≥75%	Total
All	22,593	59,936	62,560	62,933	63,257	63,421
Protein coding	1,581	18,484	19,591	19,769	19,906	19,968
Non-coding	21,012	41,452	42,969	43,164	43,351	43,453

**Table 5:** Gene annotation summary.

Gene category	Gene Biotype	CHM13	KSA001	Count of unmapped
Protein-coding	protein coding	19,969	19,968	1
Non-coding RNA	lncRNA	17,482	17,482	0
	miRNA	2,045	2,223	20
	misc RNA	2,224	2,221	3
	Mt rRNA	3	3	0
	Mt tRNA	29	29	0
	ribozyme	8	8	0
	rRNA	1,007	1,007	0
	rRNA pseudogene	506	503	3
	scaRNA	48	48	0
	scRNA	2	2	0
	snoRNA	945	944	1
	snRNA	1,886	1,883	3
	sRNA	5	5	0
	TEC	1,341	1,341	0
	vault RNA	1	1	0
Pseudogenes	pseudogene	18	15	3
	polymorphic pseudogene	50	50	0
	processed pseudogene	10,769	10,764	5
	transcribed processed pseudogene	551	550	1
	transcribed unitary pseudogene	138	137	1
	transcribed unprocessed pseudogene	941	941	0
	translated processed pseudogene	2	2	0
	translated unprocessed pseudogene	1	1	0
	unitary pseudogene	98	98	0
	unprocessed pseudogene	2,725	2,723	2
Immunoglobulin/T-cell receptor	IG C gene	15	15	0
	IG D gene	10	0	10
	IG C pseudogene	9	10	0
	IG J gene	18	8	10
	IG J pseudogene	3	3	0
	IG pseudogene	1	1	0
	IG V gene	148	148	0
	IG V pseudogene	216	214	2
	TR C gene	7	7	0
	TR J gene	80	73	7
	TR J pseudogene	4	4	0
	TR V gene	108	108	0
	TR V pseudogene	33	33	0
Unknown	StringTie	48	48	0
total		63,494	63,421	73

Counts of CHM13 genes (without chrY) mapped by Liftoff to the KSA001 genome assembly.

We further compare KSA001 with CHM13 and GRCh38 in two ways. First, we perform variant calling of short reads derived from sequencing KSA001, using CHM13 and GRCh38 as reference genomes, and report the number of variants in KSA001 when aligned against both the reference genomes (Table 6). Consistent with previously reported results^9^, we observe a substantially lower number of variants called when using CHM13 as a reference compared to using GRCh38.

**Table 6:** Variant call statistics for KSA001 Illumina reads aligned to GRCh38 and CHM13.

	Total Number of variants	SNPs	Indels
**KSA001 aligned to GRCh38**	5,337,778	4,317,999	1,115,246
**KSA001 aligned to CHM13**	4,888,875	3,934,939	1,036,797

Comparison between numbers of genes and transcripts in CHM13 and KSA001.

**Fig. 1:**
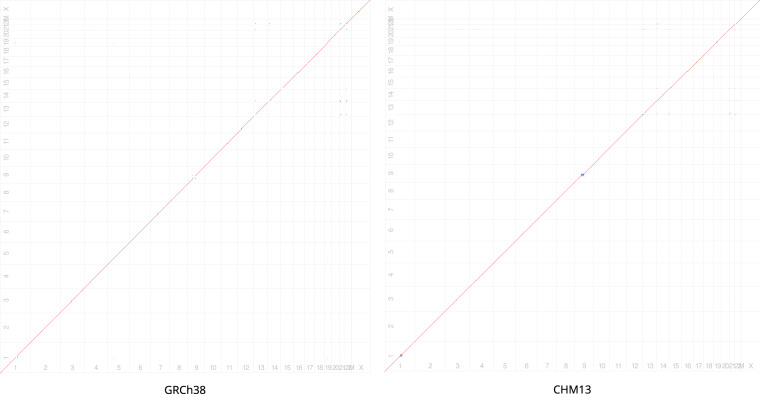
Harr plot of KSA001 aligned with GRCh38 (left) and CHM13 (right).

Second, we aligned KSA001 to CHM13 and GRCh38 using Minimap2 (v2.24-r1122) and used the call command within paftools.js to call variants. Table 7 provides summary statistics of the variants. Figure 1 depicts a Harr plot of the assemblies (Harr plots were generated using minidot from miniasm, v0.3-r179^44^).

**Table 7:** Comparison of KSA001 with CHM13 and GRCh38 based on assembly-to-assembly alignment.

	SNPs/Indels			SVs	
	SNPs	Insertion	Deletion	Insertion	Deletion
**Alignment of** KSA001 **and** CHM13	4,012,344	379,878	358,464	13,541	13,467
**Alignment of** KSA001 **and** GRCh38	3,463,231	373,606	353,703	7,539	12,523

SNPs: Single nucleotide polymorphisms, Indels: insertion or deletion, SV: Structural Variants.

Table 8 shows the completeness of the chromosomes and the number of basepairs in each chromosome of KSA001 in comparison to the CHM13 v2.0 assembly. Overall, the numbers of base-pairs in each chromosome of KSA001 are comparable to CHM13. Also, we report base-level quality (QV) scores for the KSA001 assembly for each chromosome and the entire assembly. QV score represents a log-scaled probability of error for the consensus base calls. Maternal and paternal haplotypes of KSA001 achieve a quality of 68.79 and 64.43 with some of the chromosomes reaching a QV score of almost 80. The merged haploid genome has a QV score of 72.97.

**Table 8:** Completeness of chromosomes of KSA001 compared to CHM13 and QV scores.

Chromosome	CHM13	KSA001.mat	QV.mat	KSA001.pat	QV.pat	KSA001	QV
chr1	248,387,328	246,893,779	72.66	248,515,191	68.87	249,555,359	72.66
chr2	242,696,752	242,046,071	67.91	242,632,705	75.97	243,650,403	75.97
chr3	201,105,948	201,462,031	75.93	200,338,375	77.26	200,901,366	77.26
chr4	193,574,945	191,676,519	76.81	191,977,661	76.28	193,119,457	76.81
chr5	182,045,439	181,678,762	70.83	182,455,576	58.06	182,340,321	70.83
chr6	172,126,628	171,636,591	77.00	171,747,260	73.48	172,212,606	77.00
chr7	160,567,428	160,688,708	74.99	160,686,623	78.21	161,491,582	78.21
chr8	146,259,331	145,082,495	71.28	145,751,733	78.73	146,283,325	78.73
chr9	150,617,247	142,392,075	69.80	137,639,207	59.85	144,206,915	69.80
chr10	134,758,134	137,124,436	79.28	136,124,862	75.99	137,487,260	79.28
chr11	135,127,769	134,887,184	79.08	135,515,747	67.33	135,526,054	79.08
chr12	133,324,548	133,634,782	75.29	133,706,131	61.20	137,511,954	75.29
chr13	113,566,686	113,178,117	70.10	109,813,855	60.32	114,155,372	70.10
chr14	101,161,492	98,200,010	71.78	104,568,253	75.09	104,854,739	75.09
chr15	99,753,195	95,263,998	62.42	97,348,991	72.54	100,665,885	72.54
chr16	96,330,374	91,348,093	77.78	84,634,195	74.86	91,858,333	77.78
chr17	84,276,897	83,888,424	73.11	84,257,549	76.14	84,644,139	76.14
chr18	80,542,538	80,057,793	69.88	79,675,856	55.70	80,394,818	69.88
chr19	61,707,364	63,671,480	63.93	64,545,147	65.98	66,377,225	65.98
chr20	66,210,255	66,487,749	76.83	66,499,802	79.99	66,944,161	79.99
chr21	45,090,682	43,459,253	75.13	42,689,246	75.21	43,720,397	75.21
chr22	51,324,926	42,716,621	65.58	49,396,945	66.70	49,931,329	66.70
chrX	154,259,566	154,358,968	68.79	153,509,181	65.26	154,680,412	68.79
chrM	16,569	16,567	49.39	0	0.00	16,567	49.39
chrY	62,460,029	0	-	0	-	0	-
Total	3,117,292,070	3,021,850,506	68.79	3,024,030,091	64.43	3,062,529,979	72.97

### Pathogenic variants in KSA001

We aligned KSA001 to GRCh38 and called the variants using minimap2^24^ assembly-to-assembly alignment strategy. We screened the ClinVar^45^ database and found that KSA001 is carrying four variants (ClinVar accessions VCV000360644.10, VCV000298183.17, VCV000802557.8, and VCV000293285.5) that are reported in the database as “Conflicting classifications of pathogenicity” and none of “Pathogenic” or “Likely pathogenic” variants.

### Using KSA001 in variant calling workflows

Saudi Arabia, and the Middle East and North Africa (MENA) region, have a higher burden of genetic diseases due to consanguinity^46^, the accurate calling of rare genomic variants is particularly important for the diagnosis of Mendelian disorders which are more prevalent in the region. Identifying functionally important structural variants (SVs) is more challenging as population-specific SVs are not included in reference genomes. We have identified thousands of SNPs, Indels, and SVs with a size reaching up to 102,752 bp in KSA001 compared to CHM13 (Table 7). While a single genome is not a sufficient representation of genetic variation within a population, KSA001 will likely contain several common variants from the Saudi population; including these variants in a reference used for variant calling has the potential to provide more accurate alignments of sequencing reads, and can make variant calling more efficient and specific to disease-causing variants.

The majority of genomic samples, in particular in a clinic, are still processed using short-read sequencing methods. To test whether KSA001 can be used for and potentially improve variant calling in Saudi individuals, we utilized a public genome sequence from a Saudi individual (SRR27002256^47^) sequenced on an Illumina NovaSeq 6000.

We aligned the Illumina reads to KSA001, GRCh38, and CHM13 using BWA-MEM (v0.7.17)^48^. We then sorted and indexed the alignment files using Picard (v2.20.4)^49^, and marked duplicate reads using the same tool. To reduce systematic errors in base quality scores caused by sequencers, we performed base quality score recalibration (BQSR) using GATK (v4.1.2.0)^49^ by first building a covariation model and then applying it to adjust the quality scores based on the model. For variant calling, we used HaplotypeCaller within GATK to call SNPs and small indels through a local *d*e *novo* assembly of haplotypes in specific regions. As part of this process, we also generated a number of common resources that transfer information from CHM13 to KSA001. Table 9 shows a summary of the number of variants called on the Saudi individual we are using. We identify fewer average number of variants when using KSA001 and CHM13 compared to using GRCh38, indicating that KSA001 captures major alleles in the Saudi population better than GRCh38; the number of variants identified using KSA001 as reference is similar to (although slightly more than) the number of variants identified using CHM13.

**Table 9:** Variant calling statistics for the Saudi donor (SRR27002256), using Illumina reads aligned to KSA001, CHM13, and GRCh38.

	Total Number of variants	SNPs	Indels	Homozygous variants	Heterozygous variants
**KSA001**	4,632,600	3,756,816	875,784	1,413,245	2,552,866
**CHM13**	4,525,713	3,671,251	854,462	1,306,039	2,562,750
**GRCh38**	4,928,855	4,012,232	916,623	1,595,018	2,594,096

Furthermore, we repeated the variant calling workflow using Illumina reads of the mother (SRR29122519^40^) and father (SRR29055922^50^) of KSA001 (Table 10). We identify considerably fewer variants of all types using KSA001 as a reference compared to CHM13 and GRCh38, with the exception of heterozygous variants using reads from the father. We also find fewer variants when aligning reads from the mother compared to the father which is consistent with the fact that approximately 2/3 of the haploid KSA001 genome is derived from the maternal haplotype.

**Table 10:** Variant calling statistics for the mother (SRR29122519) and father (SRR29055922) of KSA001, using Illumina reads aligned to KSA001, CHM13, and GRCh38.

Sample	Reference	Variants	SNPs	Indels	Homozygous	Heterozygous
Mother	KSA001	3,844,863	3,137,864	706,999	575,836	2,718,732
	CHM13	4,609,105	3,776,510	832,595	1,204,406	2,751,851
	GRCh38	4,979,364	4,089,298	890,066	1,474,714	2,757,196
Father	KSA001	4,446,695	3,615,255	831,440	816,030	2,733,448
	CHM13	4,647,609	3,762,777	884,832	1,281,940	2,693,290
	GRCh38	5,012,366	4,075,934	936,432	1,525,662	2,726,041

Additionally, we performed variant calling against the same three reference genomes using short reads from individuals of different ethnicities: an East Asian Han Chinese female (SRR1295554^51^), a European British male (SRR1291026^52^), an American Peruvian male (SRR1295426^53^), and a Middle Eastern Bedouin male (ERR757831^54^) (Table 11). In terms of KSA001 as a reference, we find an overall lower number of variants when aligning reads of the Han Chinese individual, also the only female compared. We also find that using CHM13 as a reference resulted in the lowest number of variants across the different ethnicities, with the exception of the Peruvian individual, which had slightly less variants when aligned to GRCh38.

**Table 11:** Variant calling statistics for individuals of varying ethnicities using Illumina reads aligned to KSA001, CHM13, and GRCh38.

Sample	Reference	Variants	SNPs	Indels	Homozygous	Heterozygous
Han Chinese	KSA001	4,659,795	3,891,211	768,584	1,591,064	2,323,911
	CHM13	4,464,929	3,729,342	735,587	1,478,926	2,305,843
	GRCh38	4,824,298	4,043,873	780,425	1,709,181	2,341,850
British	KSA001	4,989,584	4,155,236	834,348	1,469,535	2,523,526
	CHM13	4,438,021	3,685,963	752,058	1,300,352	2,451,308
	GRCh38	4,868,354	4,053,247	815,107	1,608,939	2,477,185
Peruvian	KSA001	5,235,296	4,458,182	777,114	1,682,902	2,775,561
	CHM13	5,213,319	4,441,326	771,993	1,690,479	2,770,716
	GRCh38	5,213,056	4,443,981	769,075	1,669,264	2,773,262
Bedouin	KSA001	5,057,761	4,320,790	826,971	1,464,537	2,642,526
	CHM13	4,560,734	3,802,390	758,344	1,343,928	2,568,971
	GRCh38	4,945,775	4,132,974	812,801	1,604,459	2,605,295

The individuals are an East Asian Han Chinese (SRR1295554), a European British (SRR1291026), an American Peruvian (SRR1295426), and a Middle Eastern Bedouin (ERR757831).

## Data Availability

Source code and additional resources are available at https://github.com/bio-ontology-research-group/KSA001.
